# Bioprospecting of Sea Anemones (Cnidaria, Anthozoa, Actiniaria) for β-Defensin-like α-Amylase Inhibitors

**DOI:** 10.3390/biomedicines11102682

**Published:** 2023-09-30

**Authors:** Daria Popkova, Nadezhda Otstavnykh, Oksana Sintsova, Sergey Baldaev, Rimma Kalina, Irina Gladkikh, Marina Isaeva, Elena Leychenko

**Affiliations:** G.B. Elyakov Pacific Institute of Bioorganic Chemistry, Far Eastern Branch, Russian Academy of Sciences, 159, Pr. 100 let Vladivostoku, Vladivostok 690022, Russia; daria.vladipo@yandex.ru (D.P.); chernysheva.nadezhda@gmail.com (N.O.); oksana.sintsova@uib.no (O.S.); baldaevsergey@gmail.com (S.B.); kalinarimma@gmail.com (R.K.); irinagladkikh@gmail.com (I.G.); issaeva@gmail.com (M.I.)

**Keywords:** diabetes mellitus, bioprospecting, α-amylase inhibitors, mucins, Cnidarians

## Abstract

Diabetes mellitus is one of the most serious diseases of our century. The drugs used are limited or have serious side effects. The search for new sources of compounds for effective treatment is relevant. Magnificamide, a peptide inhibitor of mammalian α-amylases, isolated from the venom of sea anemone *Heteractis magnifica,* can be used for the control of postprandial hyperglycemia in diabetes mellitus. Using the RACE approach, seven isoforms of magnificamide were detected in *H. magnifica* tentacles. The exon–intron structure of magnificamide genes was first established, and intron retention in the mature peptide-encoding region was revealed. Additionally, an α-amylase inhibitory domain was discovered in the mucins of some sea anemones. According to phylogenetics, sea anemones diverge into two groups depending on the presence of β-defensin-like α-amylase inhibitors and/or mucin-inhibitory domains. It is assumed that the intron retention phenomenon leads to additional diversity in the isoforms of inhibitors and allows for its neofunctionalization in sea anemone tentacles. Bioprospecting of sea anemones of the order *Actiniaria* for β-defensin-like α-amylase inhibitors revealed a diversity of inhibitory sequences that represents a starting point for the design of effective glucose-lowering drugs.

## 1. Introduction

Diabetes is one of the fastest-growing chronic diseases, and it is one of the top 10 causes of death in the world. For this reason, it is one of the largest public health concerns that is approaching a global epidemic rate [1]. According to the WHO, 536.6 million people aged 20–79 worldwide suffer from diabetes (data in 2021), and this number is expected to increase to 783.2 million in 2045 [2]. Previously, it was thought that the prevalence of diabetes was increasing faster in low- to middle-income countries [3], but new data confirm that the greatest relative increase in the prevalence of diabetes between 2021 and 2045 is expected to occur in middle-income countries (21.1%) compared to high- (12.2%) and low-income (11.9%) countries. Moreover, in recent years, the number of cases among children and adolescents has skyrocketed [2].

Strict control of postprandial hyperglycemia is one of the most important tasks in the treatment of type 1 (T1DM) and type 2 diabetes mellitus (T2DM), as well as the prevention of the development of T2DM in people with insulin resistance and impaired glucose tolerance. The inhibition of α-glucosidases, in particular α-amylases, can effectively control the level of postprandial glucose in the blood, preventing the occurrence of side effects associated with glucose toxicity [4]. Currently, there are three oligosaccharide inhibitors of α-glucosidases that are used as pharmacological drugs: miglitol (Glyset™), voglibose (Voglib™), and acarbose (Precose™ or Glucobay™). The first two compounds inhibit α-glucosidases and have no significant effects on α-amylase, while acarbose inhibits both classes of enzymes. Thus, all three drugs prevent the complete hydrolysis of complex carbohydrates; however, the oligosaccharides formed during hydrolysis and the inhibitors themselves can be fermented by intestinal bacteria, which leads to diarrhea and flatulence. In addition, these inhibitors are well absorbed by body tissues, which is an undesirable side effect, since the action of these drugs should be limited to the intestine [5,6,7]. All of these compounds demonstrate modest inhibitory activity, and a sufficiently large dosage is required in order to achieve the effect. This is accompanied by moderate side effects, mainly from the gastrointestinal tract. It is expected that selective inhibitors of α-amylases will have fewer side effects and greater efficacy.

Recently, two inhibitors of α-amylases, helianthamide and magnificamide, 44 a.a., have been isolated from the venom of sea anemones *Stichodactyla helianthus* [8,9] and *Heteractis magnifica* [10,11], respectively. Helianthamide was found to inhibit human and porcine pancreatic α-amylases with Ki 0.01 and 0.1 nM, respectively [8], while magnificamide inhibited porcine pancreatic and human salivary α-amylases with Ki 0.17 and 7.7 nM, respectively [11]. Since helianthamide and magnificamide belong to the β-defensin family, which is widely distributed in animals, and have a compact fold, their immunogenicity is expected to be minimal. Moreover, these peptides are resistant to proteolysis, prolonged exposure to low pH values, and high temperature, which demonstrates the possibility of their use as oral hypoglycemic agents for the control of postprandial hyperglycemia [8].

Currently, nothing is known about the function, diversity, and occurrence of α-amylase inhibitors among other sea anemones. It is possible that these molecules regulate the activity of their own α-amylases and can possibly participate in feeding and defense.

There are already many genomic and transcriptome resources for Cnidarians, as well as sequence data for public use that allow for the bioprospecting of potential pharmacological compounds. Thus, β-defensins in general, and α-amylase inhibitors in particular, represent a valuable class of biologically active compounds, but to date, only a limited number of peptides of the class have been described [12]. Herein, we first studied the diversity of α-amylase inhibitors in sea anemones transcriptomes as well as determined magnificamide-encoding transcripts and genes in *H. magnifica*. We identified the intron retention phenomenon in the α-amylase inhibitor sequences. Moreover, the α-amylase inhibitor sequence might be included as a domain in sea anemones mucins. Data on the inhibitor sequence’s diversity may determine the structural patterns which need to be applied in order to design effective glucose-lowering drugs.

## 2. Materials and Methods

### 2.1. Collection and Identification of Sea Anemone Samples

The samples of sea anemone *Heteractis magnifica,* designated as 91, 116, and 146, were collected from the South China Sea (Gulf of Siam) near Nam Du Islands, (9.71 N 104.41 E, 9.69 N 104.38 E, and 9.68 N 104.23 E), Vietnam, in 2010 and 2018, respectively, during a marine expedition aboard the research vessel Academic Oparin. The sea anemones’ tentacles (91, 116, 146) and body pieces (146) were placed into IntactRNA fixed solution (Evrogen JSC, Moscow, Russia) and stored at −20 °C before use. The identity of the species was confirmed by phylogenetic analysis of 18S rRNA, COI, and ITS sequences, as was reported recently for an *H. magnifica* sample [13].

The study was conducted according to the guidelines of the Convention on Biological Diversity and approved by the Ethics Committee of the G.B. Elyakov Pacific Institute of Bioorganic Chemistry (Vladivostok, Russia, Protocol No. 0037. 12 March 2021).

### 2.2. cDNA Determination of Magnificamide Sequences

Total RNA was isolated from the IntactRNA fixed samples of *H. magnifica* using ExtractRNA solution (Evrogen JSC, Moscow, Russia). Full-length-enriched cDNA libraries were prepared using a Mint cDNA synthesis Kit (Evrogen JSC, Moscow, Russia).

Two degenerate primers, InAmy1b and InAmy2b (Table S1), designed on the N- and C-terminal amino acid sequences of magnificamide [10], were used for PCR amplification of cDNA libraries. The PCR products were analyzed by gel electrophoresis, purified, and cloned in pTZ57R/T vector using an InsTAclone PCR Cloning Kit (Thermo Fisher Scientific, Waltham, MA, USA) and *Escherichia coli* BL21(DE3) (Novagen, Darmstadt, Germany). PCR products from positive colonies were sequenced with M13 universal primers using the SeqStudio™ Genetic Analyzer System (Thermo Fisher Scientific, Waltham, MA, USA). The full-length *Magnificamide (MGF)*-like sequences were established through rapid amplification of cDNA ends (RACE), with gene specific and standard step-out (SO-mix) primers (Table S1) from the RACE primer set (Evrogen JSC, Moscow, Russia), according to Figure S1. The established 3′- and 5′-RACE sequences were aligned using the MEGA X software version 11.0.9 [14]. The cDNA sequences were verified by PCR amplification with the InAmy_5UTR_in_F and InAmy_3UTR_R primers (Table S1). PCR fragments were cloned, sequenced, and aligned as noted above. All PCRs were conducted with Encyclo^®^ DNA Polymerase (Evrogen JSC, Moscow, Russia). All primers were synthesized by Evrogen JSC (Moscow, Russia).

The obtained sequences were deposited into GenBank databases under the accession numbers OR141967 (magnificamide 1-1), OR141968 (magnificamide 1-2), OR141969 (magnificamide 1-3), OR141970 (magnificamide 1-4), OR141971 (magnificamide 1-5), OR141972 (magnificamide 1-6), and OR141973 (magnificamide 2-1).

### 2.3. Magnificamide Gene Structure Determination

To determine an exon–intron structure of the *MGF* gene of *H. magnifica* (isolate IRGN:W9CBD1RZL8), the WGS project (JAADYU000000000.1) and raw data from the SRA database (SRR8614882, and SRR12065068) were used. A BLAST search with the obtained cDNA sequence was performed. Matched contigs and reads were aligned using MEGA X software [14]

To validate a gene assembly, a set of primers was designed using the Vector NTI software package, version 11.0 (Invitrogen, Carlsbad, CA, USA). Genomic DNA (gDNA) was extracted from the *H. magnifica* 116 and 146 samples using the MagJET Plant Genomic DNA Kit (Thermo Fisher Scientific, Waltham, MA, USA), then amplified with following primer sets: InAmy_5UTR_out_F and InAmy_NF_full_R; InAmy_NF_sig and InAmy_Intron2_R; InAmy_NF_sig and InAmy_3UTR_R (Table S1). The whole-gene sequence was amplified using the InAmy_5UTR_out_F and InAmy_3UTR_R primers. PCR amplification, cloning, and sequencing were carried out as described in Section 2.2.

### 2.4. Detection of Intron Retention in Transcripts

*Magnificamide* cDNAs were examined for intron retention using intron-specific and flanking primers (Table S1). gDNAs (as a control) and cDNAs of the 116 and 146 samples were used as templates with following primer sets: InAmy_5UTR_in_F and InAmy_NF_full_R; InAmy_NF_sig and InAmy_insideIn2_R; and InAmy_insideIn3_F and InAmy_3UTR_R. To check the contamination of cDNAs with gDNA, two pairs of primers were designed based on the exon and intron regions of glyceraldehyde-3-phosphate dehydrogenase (GAPDH) (Table S1). A *GAPDH* gene of *H. magnifica* was assembled using TBLASTN and BLASTN with queries of GAPDH from *Exaiptasia diaphana* (KXJ17204.1, XM_021039629.1) against the databases (GJFJ00000000.1, JAADYU000000000.1, SRR8614882, and SRR12065068). The *GAPDH* assembly was validated by direct sequencing.

The obtained sequences were deposited into the GenBank databases under the accession numbers OR540620 (magnificamide_i3_v.1) and OR540621 (magnificamide_i3_v.2).

### 2.5. Alignment and Phylogenetic Analysis

A search of magnificamide homologs was performed in the GenBank and TSA (Transcriptome Shotgun Assembly Sequence) databases (Table 1) using TBLASTN (http://www.ncbi.nlm.nih.gov/BLAST, accessed on 24 June 2023). The multiple sequence alignments were performed using MEGA X software, version 11.0.9 [14], with the ClustalW algorithm. The amino acid sequence’s identity was calculated using the AlignX function of Vector NTI Advance 11.0 (Invitrogen, Carlsbad, CA, USA).

A maximum likelihood (ML) tree with 100 non-parametric bootstrap replicates was produced using the IQ-TREE web server (version 1.6.12), according to the best-fit substitution model WAG + I + G4, determined using ModelFinder [15,16].

### 2.6. Comparative Analysis of Sequences In Silico

For the comparative analysis of sequences, the NULA algorithm was applied as described in [17]. After NULA numbers were calculated using Microsoft Excel 2007, a 3D scatter plot was generated using ORIGIN 8.5 software. Homology models of peptides based on the 3D structure of helianthamide (PDB ID: 4X0N [8]) were constructed using the SWISS-MODEL web server [18]. Dipole moments were calculated using a Discovery studio 4.0 Visualizer (Accelrys Software Inc., San Diego, CA, USA), and plots were generated using ORIGIN 8.5 software.

## 3. Results

### 3.1. cDNA and Gene Structural Organization of Magnificamides

**Figure 1:**
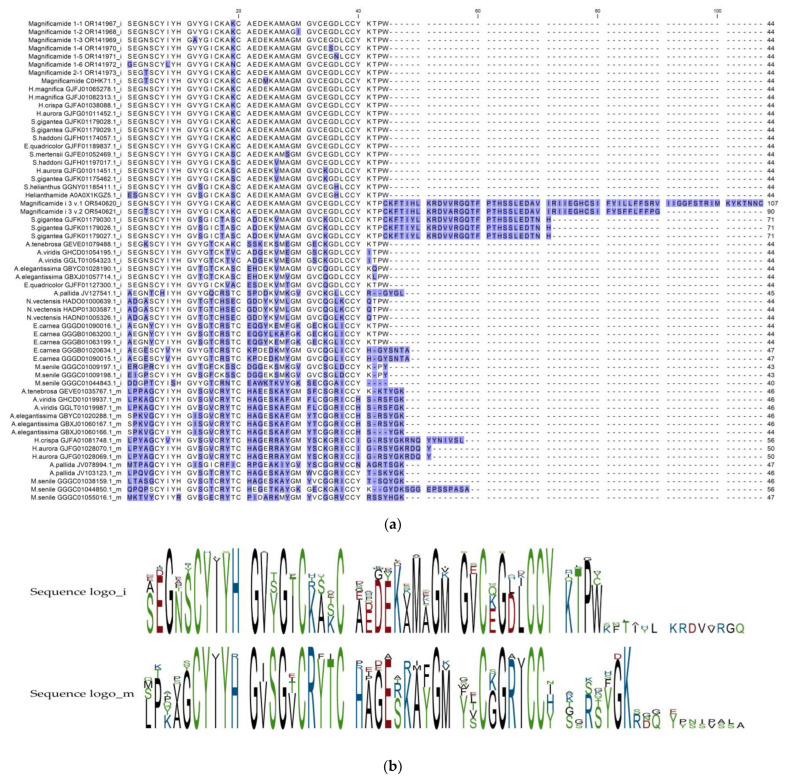
Multiple alignment (**a**) and sequence logos (**b**) for magnificamide inhibitors (i) and mucin-inhibitory domains (m) among representatives of the order *Actiniaria* (sea anemones *sensu stricto*), predicted using TBLASTN. Alignments were generated using the CLC genomic workbench (CLC Bio, Qiagen, Redwood City, CA, USA).

The Step-Out RACE approach was applied to elucidate the existence of isoforms of magnificamide, an α-amylases inhibitor, which was previously isolated from *H. magnifica* [10]. The detailed strategy for finding magnificamide-encoding transcripts is shown in Figure S1. The *Magnificamide* cDNA sequence obtained by 5′- and 3′-RACE assembly consisted of 460 bp, including sequences encoding a signal peptide, a propeptide, and a mature peptide (54, 30, and 135 bp, respectively), as well as non-coding regions (Figure S2). Seven magnificamide isoforms were deduced based on the sequencing of 77 clones; two isoforms—magnificamide 1-1 from the *H. magnifica* 116 sample and magnificamide 2-1 from the *H. magnifica* 91 sample—were found with 39 (50.6%) and 33 (42.9%) copies, respectively. Other sequences (magnificamides 1-2, 1-3, 1-4, 1-5, and 1-6 from the *H. magnifica* 116 sample) were found as single gene copies. All magnificamide isoforms had single amino acid substitutions in the mature peptides, while the signal peptide and propeptide sequences were identical (Figure 1).

**Figure 2:**
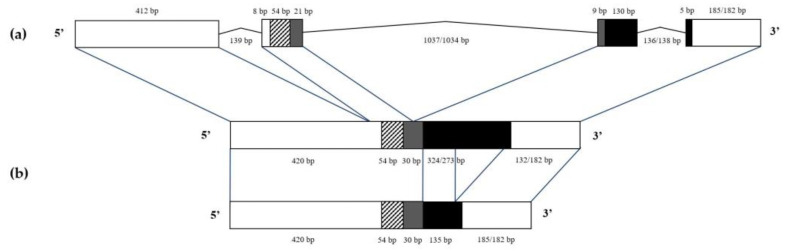
Magnificamide cDNA and gene structures. The gene structure (**a**) was predicted based on WGS and SRA data analysis and confirmed by PCR with gene-specific primers. Two different splice variants of magnificamide cDNAs, (**b**) obtained as a result of retention or removal of the third intron, were detected by RACEs and cDNA sequencing. White and grey boxes represent untranslated regions (5′- and 3′-UTRs) and propeptides, respectively. The diagonally shaded box indicates signal peptides, and black boxes indicate mature magnificamide sequences. The sequence lengths of magnificamide 1-1 and magnificamide 2-1 are shown by a slash.

To date, nothing is known about the gene structure of α-amylase inhibitors from sea anemones. In the present study, the gene structure was predicted in silico through mapping of contigs and corresponding raw reads of the *H. magnifica* genome (isolate IRGN:W9CBD1RZL8, genome assembly ASM1176337v2) onto a full length cDNA sequence, then confirmed with gene-specific primers. According to the obtained data, the gene contains three introns located inside the 5′-UTR region, as well as propeptide- and mature peptide-encoding sequences (Figure 2a).

Sequence analysis showed that the introns had conventional borders with GT and AG dinucleotides, located at the 5′ and 3′ ends as donor and acceptor splicing sites, respectively. The gene parts encoding a mature peptide matched with the sequences of major isoforms, magnificamide 1-1 and magnificamide 2-1. The magnificamide 1-1 and magnificamide 2-1 gene sequences verified by PCR amplification were deposited at GenBank NCBI under the accession numbers OR499880 and OR499881, respectively. It was surprising that the third intron was present in the some sequences of magnificamide (Figure 2b).

### 3.2. Intron Retention Phenomenon in Magnificamide cDNAs

To confirm the reliability of *in silico* assembly of the magnificamide gene, PCR with gene-specific primers (Table S1) was performed with the gDNA and cDNA of *H. magnifica* 116 and 146 samples. Intron retention was tested with intron-specific primers (Table S1).

We applied the methodology described by Moran et al. [19], according to which the *gapdh* gene serves as a control determining whether the cDNA is contaminated with traces of gDNA; the primers were directed to *gapdh* exon–intron boundaries and the intronic region (Figure S3). The *gapdh* intron was absent in the control samples, confirming the purity of cDNA preparations. The third intron was found in both 116 and 146 tentacle cDNA samples (Figure S3b). 

A detailed sequence analysis showed that the third intron interrupted a codon-encoding tryptophan, the last residue, but it was in-frame with a magnificamide gene. Thus, magnificamides 1-1 and 2-1 could be extended by 63 and 46 additional amino acid residues, respectively. However, after correct splicing according to the GT-AG rule, both the Trp and Stop codons were recovered. Obviously, intron retention is a phenomenon leading to the diversity of magnificamide isoforms in the tentacles.

Interestingly, intronic sequences were found in some magnificamide-like transcripts among other sea anemones under BLASTN search; each such transcript retained one or two out of three introns. Retention of one of the three introns was detected in *Stichodactyla gigantea* (GJFK01179028.1, GJFK01179030.1), *Stichodactyla haddoni* (GJFH01174057.1), and *H. magnifica* (GJFJ01065278.1, GJFJ01065279.1), whereas some transcripts of *S. gigantea* (GJFK01179027.1, GJFK01179026.1) and *S. haddoni* (GJFH01174056.1) tentacles displayed retention of two out of three introns.

### 3.3. Search for Sea Anemone α-Amylase Inhibitor Homologues

To investigate the presence and diversity of α-amylase inhibitor homologues in other sea anemones, magnificamide 1-1 cDNA was subjected to a BLAST search against the TSA database of the order *Actiniaria* (taxid:6103). As a result, the homologous sequences in the *H. magnifica* (GJFJ00000000.1), *H. crispa* (GJFA00000000.1), *H. aurora* (GJFG00000000.1), *E. quadricolor* (GJFF00000000.1), *Stichodactyla mertensii* (GJFE00000000.1), *S. haddoni* (GJFH00000000.1), *Stichodactyla gigantea* (GJFK00000000.1), and *Stichodactyla helianthus* (GGNY00000000.1) transcriptomes were identified.

**Figure 3:**
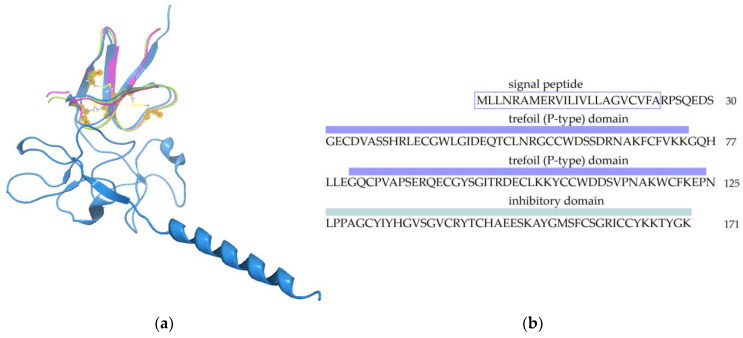
Sea anemone mucin structure. (**a**) Overlay of the spatial structures of inhibitory domains was performed using the CLC genomic workbench (CLC Bio, Qiagen): blue—*A. tenebrosa* mucin (A0A6P8IY78 UniProtKB, 171 aa); green—magnificamide 1-1 (44 aa); pink—helianthamide (A0A0 × 1KGZ5 UniProtKB, 44 aa). (**b**) Domain organization of *A. tenebrosa* mucin: violet—trefoil domains (44 aa); turquoise—inhibitory domain (46 aa).

A search for homologues of magnificamide (C0HK71 UniProtKB, 44 aa), performed against the GenBank database using TBLASTN search, revealed 34 sequences with 40–100% identity between them (Figure 1a). It was found that α-amylase inhibitor sequences are included into some sea anemone mucins as a domain. To search for α-amylase inhibitory domains in sea anemone mucins, *Actinia tenebrosa* mucin (A0A6P8IY78 UniProtKB, 171 aa) was subjected to a TBLASTN search against the TSA database (Table 1). As a result, 14 mucin sequences containing the inhibitory domain, with 34–45% identity of magnificamide (C0HK71 UniProtKB, 44 aa), were revealed (Figure 1b). It was shown that the α-amylase binding site (YIYH) predicted for helianthamide [8] was identical in all discovered sequences (except three), as well as in most domains of mucins. The sequence identity between magnificamide and α-amylase inhibitory domains of mucins did not exceed 45%, while their predicted 3D structures were similar (Figure 3).

**Figure 4:**
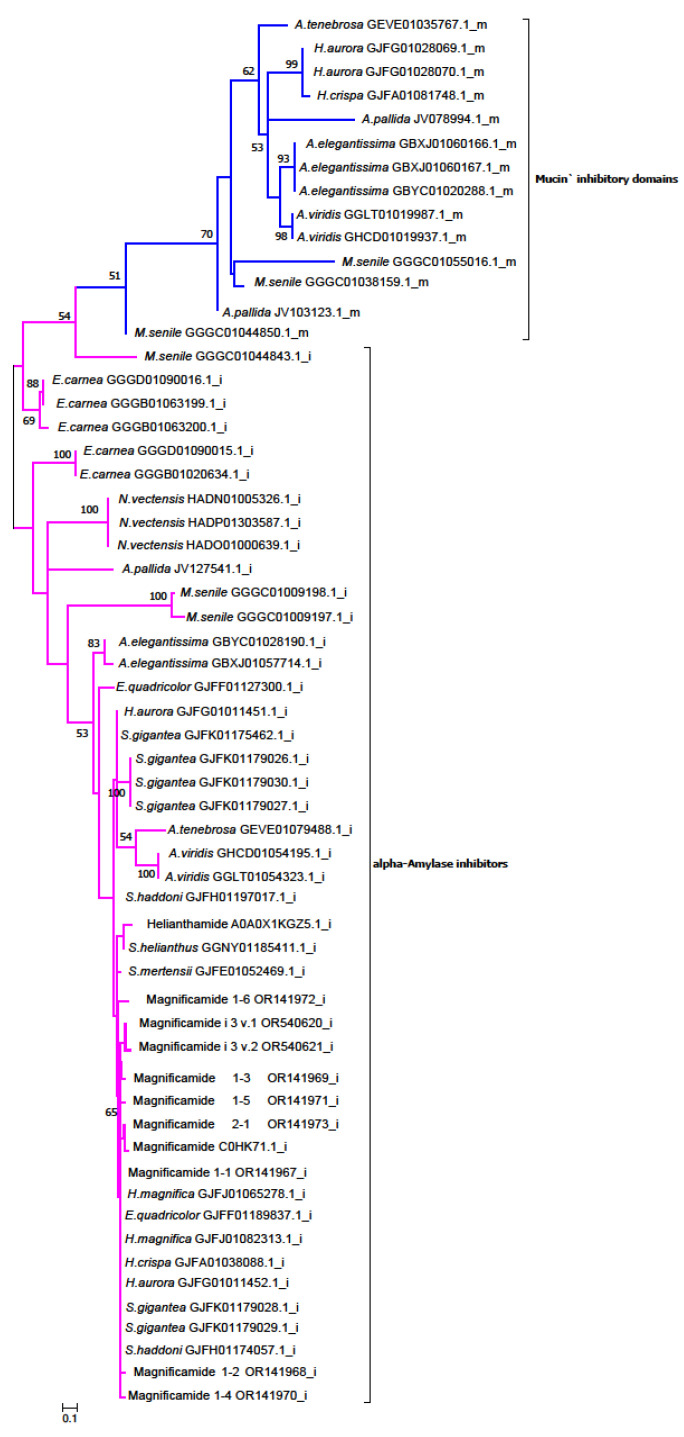
The unrooted phylogenetic tree of α-amylase inhibitors (helianthamide and magnificamide) and their homologs (i) and mucin-inhibitory domains (m) from sea anemones. Numbers next to branches represent bootstrap support. The ML tree was produced by the IQ-TREE web server (version 1.6.12), according to the WAG + I + G4 model. Β-defensin-like inhibitor branches are shown in pink, and mucin-inhibitory domains are shown in blue.

To determine the evolutionary relationships between α-amylase inhibitors and mucin α-amylase inhibitory domains of sea anemones, the discovered sequences were used to construct a ML phylogenetic tree (Figure 4). As a result of the phylogenetic analysis, two separate groups were revealed. One of them, which was well-formed, consisted of mucin-inhibitory domain sequences, while the other group, which included α-amylase inhibitors, was larger, with low bootstrap support of the branches. Interestingly, α-amylase inhibitors from phylogenetically distant sea anemones (*H. crispa* (GJFA01038088.1), *H. aurora* (GJFG01011452.1), and *E. quadricolor* (GJFF01189837.1)) fell into the magnificamide/helianthamide clade (up to 100% identity).

Furthermore, sea anemones can be divided into two groups according to the presence of β-defensin-like α-amylase inhibitors and/or mucin-inhibitory domains. The non-mucin group includes representatives of the family *Stichodactylidae* (*H. magnifica*, *S. helianthus*, *S. mertensii*, *S. gigantea*, *S. haddoni*), as well as the families *Edwardsiidae* (*Nematostella vectensis, Edwardsiella carnea*) and *Actiniidae* (*E. quadricolor*), whose transcriptomes contain only β-defensin-like inhibitors. However, other representatives of the family *Actiniidae* (*A. tenebrosa*, *Anemonia*, *Anthopleura elegantissima*) belong to the mucin group, which includes both β-defensin-like inhibitors and mucins with α-amylase inhibitory domains. This group also includes two representatives of the genus *Heteractis* (*H. crispa*, *H. aurora*), as well as representatives of the superfamily *Metridioidea* (*Metridium senile*, *Aiptasia pallida*).

**Figure 5:**
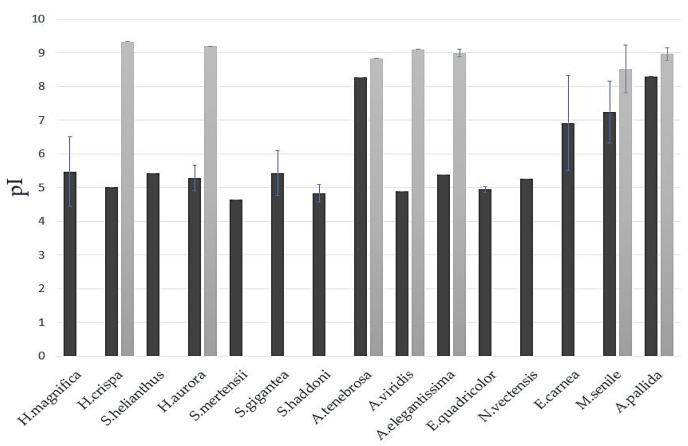
Histogram of the isoelectric point (*pI*) distribution of β-defensin-like inhibitors (black) and mucin-inhibitory domains (grey) among representatives of sea anemones.

Almost all β-defensin-like inhibitors in the non-mucin group have an isoelectric point (*pI*) of 4.5–5.7 (except for *E. carnea* ones, *pI* ~ 6.9), while all mucin-inhibitory domains exhibit *pI* values of 8.5–9.3 (Figure 5). The inhibitors found in these species can be divided into two groups: *pI*~5 (from *H. crispa*, *H. aurora*, *A. viridis*, and *A. elegantissima*) and ~ 7.5 (from *A. tenebrosa*, *M. senile*, and *A. pallida*).

**Figure 6:**
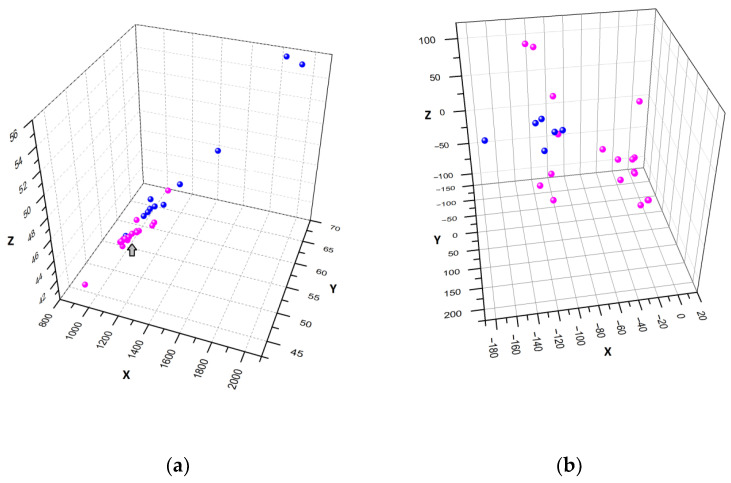
Three-dimensional representation of α-amylase inhibitors (purple spheres) and mucin-inhibitory domains (blue spheres) from sea anemones. (**a**) A 3D scatter plot of sequence coordinates prepared by the NULA algorithm. Magnificamides’ locations are shown by the arrow. (**b**) A 3D scatter plot of the coordinates of the superimposed molecules’ dipole moments.

To estimate the structural features of the analyzed sequences, the Number of Lareo and Acevedo algorithm (NULA) [17] and distribution of dipole moment direction (as a total indicator of molecule electrostatic properties) were used. It has previously been reported that sea anemone toxins modulating sodium and potassium channels were successfully clustered using the NULA algorithm [20]. After the conversion of each sequence into a set of orthogonal coordinates, a 3D scatter plot was obtained (Figure 6a). It can be seen that magnificamides were distinctly grouped with each other and with other α-amylase inhibitors, while sequences of mucin inhibitory domains were localized adjacently. The exceptions were a mucin-inhibitory domain from *A. elegantissima* (GBXJ01060166.1), which colocalized with magnificamides, and α-amylase inhibitors from *E. carnea* (GGGD01090015.1 and GGGD01090016.1) and *A. tenebrosa* (GEVE01079488.1), which were mixed up with mucin domains. A similar distribution was obtained from the comparison of the dipole moment direction of peptide molecules (Figure 6b). In general, there were two clusters. One cluster included implicitly grouped mucin-inhibitory domains with several α-amylase inhibitors, and another cluster was formed only by α-amylase inhibitors, including magnificamides.

## 4. Discussion

One of the therapeutic approaches for the treatment of diabetes is targeted α-amylases inhibition [4]. The low-molecular-weight compounds that are used for this purpose today—miglitol, voglibose, and acarbose—have undesirable side effects [5,6,7]. The search for new compounds for the prevention of postprandial hyperglycemia is relevant due to the high prevalence of this disease among the population [3,4]. Polypeptide-based drugs have recently been considered as a more effective and safer alternative to low-molecular-weight compounds [21]. However, most known protein inhibitors of α-amylases, which are bacterial polypeptides (78 aa) from *Streptomyces*, cannot be used in clinical practice due to their high immunogenicity [8]. The recently discovered sea anemone peptides helianthamide and magnificamide belong to the β-defensin family, whose representatives are widely distributed in mammals, including humans, determining their low immunogenicity. Along with the compactness and stability of the structure, which facilitates oral administration, such peptides appear to be promising as drug prototypes [8,11].

Biologically active peptides of sea anemones are known to form so-called combinatorial libraries, the number of representatives of which can reach dozens of compounds. For example, multigene families of actinoporins [22,23] and Kunitz-type peptides [24] were found in *H. magnifica* (=*H. crispa*). Here, using the RACE approach, we were able to detect *magnificamide* transcripts encoding seven isoforms of magnificamide which differed in point substitutions. Magnificamides 1-1 and 2-1 are of interest for further detailed studies as major isoforms of *H. magnifica* β-defensin-like peptides. According to the TBLASTN results, transcriptomes of other sea anemones also share a number of *magnificamide*-like transcripts. For example, three isoforms were found to be encoded by at least six genes in *S. gigantea*.

For the first time, the exon–intron structure of magnificamide genes was established; three introns with lengths of 139, 1037/1034, and 136/138 bp separated exons, including the mature peptide-encoding region. It was revealed that the third intron sequence was retained in some magnificamide transcripts from *H. magnifica* tentacles. The same transcripts which retained the third intron were observed in *S. gigantea* tentacles. Interestingly, an intron retention was characteristic of other β-defensin-like α-amylase inhibitors from sea anemones; such transcripts also retained one or two out of three introns. This phenomenon leads to a variety of isoforms of β-defensin-like α-amylase inhibitors in sea anemone tentacles.

For the first time, we discovered that some sea anemone mucins contain an α-amylase inhibitory domain in their structure. Mucins are a group of functionally characterized glycoproteins defined by the presence of PTS repeats (repetitive glycosylation sites rich in proline (P), threonine (T), and serine (S) residues). The main functions of mucins are the formation of mucous layers to lubricate various organs and the provision of a protective barrier against environmental influences [25]. Moreover, mucins form an interface with commensal and pathogenic microbes, facilitating both colonization by physiological microflora and host defense against pathogens [26]. Besides the synthesis of β-defensin-like α-amylase inhibitors in venomous secretions, some species of sea anemones are able to synthesize such peptides as domains of mucins. It has been found that the YIYH reaction site [8] is highly conserved in most sea anemones’ mucin domains. Therefore, it can be assumed that they protect their own carbohydrate components from hydrolysis by glycoside hydrolases of other marine hydrobionts.

We assume that an inhibitor with a *pI* of about 9 protects mucin, while an inhibitor with a *pI* of about 5–6 regulates α-amylase activity. Interestingly, when an intron was retained, the *pI* of magnificamide increased up to 8.5. Apparently, this is one of the ways to protect mucins in species belonging to the non-mucin group. Therefore, the intron retention phenomenon contributes to the neofunctionalization of β-defensin-like α-amylase inhibitors. Undoubtedly, this hypothesis requires further experimental verification. Thus, the discovered diversity of inhibitory sequences can serve as a starting point for the design of effective glucose-lowering drugs.

## 5. Conclusions

The bioprospecting of biologically active natural products in the early stages of drug development is a very effective tool. Genomic bioprospecting of marine organisms for novel therapies is an emerging trend that is currently gaining momentum and is based on advances in genomic/transcriptome research. Marine ecosystems represent an untapped reservoir of biodiversity with enormous potential for bioprospecting of pharmacologically active compounds. Herein, we attempted to systematically explore sea anemones of the order *Actiniaria* for β-defensin-like α-amylase inhibitors in order to elucidate the diversity of magnificamide-like peptides, which are α-amylase inhibitors, while focusing on our object of interest, the sea anemone *H. magnifica*. Our results confirm that transcriptomic data alone are not sufficient, and that additional molecular genetic studies are needed in order for us to gain a general understanding of the diversity and function of biologically active compounds. Thus, the suddenly identified β-defensin-like α-amylase inhibitor domain in sea anemone mucins may be promising for drug design.

## Figures and Tables

**Table 1:** NCBI accession numbers of the assembled transcriptomes of sea anemones from the order *Actiniaria,* used to search for magnificamide homologs.

Sea Anemone Species	Accession Number	BioProject	BioSample	Total Length, bp
*Heteractis magnifica*	GJFJ01000000	PRJNA723429	SAMN18817342	200,990,580
*Heteractis crispa*	GJFA01000000		SAMN18817339	165,422,863
*Heteractis aurora*	GJFG01000000		SAMN18817340	174,630,657
*Stichodactyla mertensii*	GJFE01000000		SAMN18817343	174,763,270
*Stichodactyla gigantea*	GJFK01000000		SAMN18817344	241,449,846
*Stichodactyla haddoni*	GJFH01000000		SAMN18817345	272,447,791
*Entacmaea quadricolor*	GJFF01000000		SAMN18817341	211,899,929
*Stichodactyla helianthus*	GGNY01000000	PRJNA464282	SAMN09080666	171,139,446
*Actinia tenebrosa*	GEVE01000000	PRJNA295912	SAMN04093377	79,258,934
*Anemonia viridis*	GHCD01000000	PRJNA448978	SAMN08913555– SAMN08913566	82,021,220
	GGLT01000000			82,153,155
*Anthopleura elegantissima*	GBYC01000000	PRJNA266623	SAMN03168566	110,645,889
	GBXJ01000000		SAMN03168566	233,919,486
*Nematostella vectensis*	HADO01000000	PRJEB13676	SAMEA3937621 SAMEA3937624 SAMEA3937637	34,622,006
	HADN01000000			118,733,569
	HADP01000000		SAMEA3937621– SAMEA3937626 SAMEA3937635– SAMEA3937637	250,709,825
*Edwardsiella carnea*	GGGB01000000	PRJNA430035	SAMN08369976	74,917,064
	GGGD01000000		SAMN08369946	121,160,303
*Metridium senile*	GGGC01000000		SAMN08365318	49,591,943
*Exaiptasia diaphana* *(syn: Aiptasia pallida)*	JV077153–JV134524	PRJNA159215	SAMN01178416	-

## Data Availability

Not applicable.

## Supplementary Materials

The following supporting information can be downloaded at: https://www.mdpi.com/article/10.3390/biomedicines11102682/s1, Figure S1: The RACE scheme for full-length cDNA sequencing determination of magnificamide-encoding genes. Figure S2: cDNA obtained by rapid amplification of cDNA ends and deduced magnificamide sequence. The exons are in uppercase letters, while the introns are in lowercase letters. The deduced amino acid residues are represented as single-letter abbreviations. The signal peptide and propeptide are in bold letters and underlined in bold, respectively. The sequence corresponding to magnificamide 1-1 is boxed. Figure S3: Agarose gel images of PCR products obtained by the detection of intron retention with appropriate primers (a) (Table S1), testing of 116 and 146 tentacle samples (lanes 1 and 3, respectively), and 146 body sample (lane 2) for the presence of a third intron retained in cDNAs (b). L—100+ bp DNA Ladder (Evrogen JSC, Moscow, Russia). Table S1: Designations and nucleotide sequences of the gene-specific primers used in the study.

## References

[1] Lin X., Xu Y., Pan X., Xu J., Ding Y., Sun X., Song X., Ren Y., Shan P.-F. (2020). Global, Regional, and National Burden and Trend of Diabetes in 195 Countries and Territories: An Analysis from 1990 to 2025. Sci. Rep..

[2] Sun H., Saeedi P., Karuranga S., Pinkepank M., Ogurtsova K., Duncan B.B., Stein C., Basit A., Chan J.C.N., Mbanya J.C. (2022). IDF Diabetes Atlas: Global, Regional and Country-Level Diabetes Prevalence Estimates for 2021 and Projections for 2045. Diabetes Res. Clin. Pract..

[3] World Health Organization Diabetes Programme. http://www.who.int/diabetes/en/.

[4] Patil S.P., Goswami A., Kalia K., Kate A.S. (2020). Plant-Derived Bioactive Peptides: A Treatment to Cure Diabetes. Int. J. Pept. Res. Ther..

[5] Scheen A.J. (2003). Is There a Role for Alpha-Glucosidase Inhibitors in the Prevention of Type 2 Diabetes Mellitus?. Drugs.

[6] Scott L.J., Spencer C.M. (2000). Miglitol: A Review of Its Therapeutic Potential in Type 2 Diabetes Mellitus. Drugs.

[7] Scheen A.J. (1997). Drug Treatment of Non-Insulin-Dependent Diabetes Mellitus in the 1990s. Drugs.

[8] Tysoe C., Williams L.K., Keyzers R., Nguyen N.T., Tarling C., Wicki J., Goddard-Borger E.D., Aguda A.H., Perry S., Foster L.J. (2016). Potent Human α-Amylase Inhibition by the β-Defensin-like Protein Helianthamide. ACS Cent. Sci..

[9] Tysoe C., Withers S.G. (2018). Structural Dissection of Helianthamide Reveals the Basis of Its Potent Inhibition of Human Pancreatic α-Amylase. Biochemistry.

[10] Sintsova O., Gladkikh I., Chausova V., Monastyrnaya M., Anastyuk S., Chernikov O., Yurchenko E., Aminin D., Isaeva M., Leychenko E. (2018). Peptide Fingerprinting of the Sea Anemone *Heteractis magnifica* Mucus Revealed Neurotoxins, Kunitz-Type Proteinase Inhibitors and a New β-Defensin α-Amylase Inhibitor. J. Proteom..

[11] Sintsova O., Gladkikh I., Kalinovskii A., Zelepuga E., Monastyrnaya M., Kim N., Shevchenko L., Peigneur S., Tytgat J., Kozlovskaya E. (2019). Magnificamide, a β-Defensin-Like Peptide from the Mucus of the Sea Anemone *Heteractis magnifica*, Is a Strong Inhibitor of Mammalian α-Amylases. Mar. Drugs.

[12] Mitchell M.L., Shafee T., Papenfuss A.T., Norton R.S. (2019). Evolution of Cnidarian *Trans*-defensins: Sequence, Structure and Exploration of Chemical Space. Proteins Struct. Funct. Bioinform..

[13] Kalina R.S., Kasheverov I.E., Koshelev S.G., Sintsova O.V., Peigneur S., Pinheiro-Junior E.L., Popov R.S., Chausova V.E., Monastyrnaya M.M., Dmitrenok P.S. (2022). Nicotinic Acetylcholine Receptors Are Novel Targets of APETx-like Toxins from the Sea Anemone *Heteractis magnifica*. Toxins.

[14] Kumar S., Stecher G., Li M., Knyaz C., Tamura K. (2018). MEGA X: Molecular Evolutionary Genetics Analysis across Computing Platforms. Mol. Biol. Evol..

[15] Kalyaanamoorthy S., Minh B.Q., Wong T.K.F., von Haeseler A., Jermiin L.S. (2017). ModelFinder: Fast Model Selection for Accurate Phylogenetic Estimates. Nat. Methods.

[16] Madeira F., Park Y.M., Lee J., Buso N., Gur T., Madhusoodanan N., Basutkar P., Tivey A.R.N., Potter S.C., Finn R.D. (2019). The EMBL-EBI Search and Sequence Analysis Tools APIs in 2019. Nucleic Acids Res..

[17] Lareo L.R., Acevedo O.E. (1999). Sequence Mapping in a Three-Dimensional Space by a Numeric Method and Some of Its Applications. Acta Biotheor..

[18] Waterhouse A., Bertoni M., Bienert S., Studer G., Tauriello G., Gumienny R., Heer F.T., de Beer T.A.P., Rempfer C., Bordoli L. (2018). SWISS-MODEL: Homology Modelling of Protein Structures and Complexes. Nucleic Acids Res..

[19] Moran Y., Weinberger H., Reitzel A.M., Sullivan J.C., Kahn R., Gordon D., Finnerty J.R., Gurevitz M. (2008). Intron Retention as a Posttranscriptional Regulatory Mechanism of Neurotoxin Expression at Early Life Stages of the Starlet Anemone *Nematostella vectensis*. J. Mol. Biol..

[20] Morales L., Acevedo O., Martínez M., Gokhman D., Corredor C. (2009). Functional Discrimination of Sea Anemone Neurotoxins Using 3D-Plotting. Open Life Sci..

[21] Robinson S.D., Safavi-Hemami H. (2017). Venom Peptides as Pharmacological Tools and Therapeutics for Diabetes. Neuropharmacology.

[22] Wang Y., Yap L.L., Chua K.L., Khoo H.E. (2008). A Multigene Family of *Heteractis* Magnificalysins (HMgs). Toxicon.

[23] Leichenko E.V., Monastirnaya M.M., Zelepuga E.A., Tkacheva E.S., Isaeva M.P., Likhatskaya G.N., Anastyuk S.D., Kozlovskaya E.P. (2014). Hct-a Is a New Actinoporin Family from the *Heteractis crispa* Sea Anemone. Acta Naturae.

[24] Kvetkina A., Leychenko E., Chausova V., Zelepuga E., Chernysheva N., Guzev K., Pislyagin E., Yurchenko E., Menchinskaya E., Aminin D. (2020). A New Multigene HCIQ Subfamily from the Sea Anemone *Heteractis crispa* Encodes Kunitz-Peptides Exhibiting Neuroprotective Activity against 6-Hydroxydopamine. Sci. Rep..

[25] Linden S.K., Sutton P., Karlsson N.G., Korolik V., McGuckin M.A. (2008). Mucins in the Mucosal Barrier to Infection. Mucosal Immunol..

[26] McAuley J.L., Linden S.K., Png C.W., King R.M., Pennington H.L., Gendler S.J., Florin T.H., Hill G.R., Korolik V., McGuckin M.A. (2007). MUC1 Cell Surface Mucin Is a Critical Element of the Mucosal Barrier to Infection. J. Clin. Investig..

