# Supplementary material for: Bioprospecting of Sea Anemones (Cnidaria, Anthozoa, Actiniaria) for β-Defensin-like α-Amylase Inhibitors
Source: Biomedicines. 2023 Sep 30;11(10):2682. doi: 10.3390/biomedicines11102682 (PMC10604346; doi:10.3390/biomedicines11102682)
Supplement: Supplementary file 1 [file biomedicines-11-02682-s001.zip › biomedicines-2616804-supplementary.pdf]

Bioprospecting of Sea Anemones (Cnidaria, Anthozoa, Actiniaria) for β-Defensin-like α-amylase Inhibitors

Daria Popkova, Nadezhda Otstavnykh, Oksana Sintsova, Marina Isaeva, Sergey Baldaev, Irina Gladkikh, and Elena Leychenko*

G.B. Elyakov Pacific Institute of Bioorganic Chemistry, Far Eastern Branch, Russian Academy of Sciences, 159, Pr. 100 let Vladivostoku, 690022, Vladivostok and Russian Federation; daria.vladipo@yandex.ru (D.P.); chernysheva.nadezhda@gmail.com (N.O.); oksana.sintsova@uib.no (O.S.); baldaevsergey@gmail.com (S.B.); irinagladkikh@gmail.com (I.G.); leychenko@gmail.com (E.L.).
*Correspondence: leychenko@gmail.com (E.L.)

Table S1. Designations and nucleotide sequences of the gene-specific primers used in the study.

Designation of primer	Nucleotide sequence of primer (5′→3′)	Primer localization
Determination of the full-length magnificamide-like cDNA sequence		
InAmy1b	ATGYTAYATHTAYCAYGGYGT	6-CYIYHG-11
InAmy2b	TGCTGYTAYAARACNCCNTGGT	38-CCYKTPW-44
InAmyNF	CGGGATATGCAAGGCTAAGT	14-GICKAK-19
InAmyCF	GGTGTGTGTGAAGGCGAT	31-GVCEGD-36
InAmyCR_3	GTATTTCATTATCCTGGTCG	3′-untranslated
InAmyCR	ATCGCCTTCACACACACC	31-GVCEGD-36
InAmy_5UTR_in_F	ATAAACCATCAGTGGCGAAATTCT	5′-untranslated
InAmy_3UTR_R	TCTGAGTTTCAAAGGAGCCGT	3′-untranslated
Validation of the gene assembly		
InAmy_5UTR_out_F	CCAGGGACAAACTCAGGCTGT	5′-untranslated
InAmy_NF_full_R	CAAGGGCAAGACCAACCAGAAG	signal
InAmy_NF_sig	CTTCTGGTTGGTCTTGCCCTTG	signal
InAmy_Intron2_R	ATAGCACGAGTTGCCCTCAGA	mature peptide
Detection of intron retention		
InAmy_insideIn2_R	AGTRCCACACACAATCAAACGA	intron
InAmy_insideIn3_F	CAATCCATCTCAAGAGGGATG	intron
HmagGAPDH_F	CTCGTTTGTCGTGCTAGTTTGGA	exon
HmagGAPDH_R	CTTGACATCTCCCTTGAATCGGC	exon
HmagGAPDH_intron_F	ACGCTTTATCACCATCTGTTCAT	intron
HmagGAPDH_intron_R	CACCCTCCAAAGGTCCACG	intron

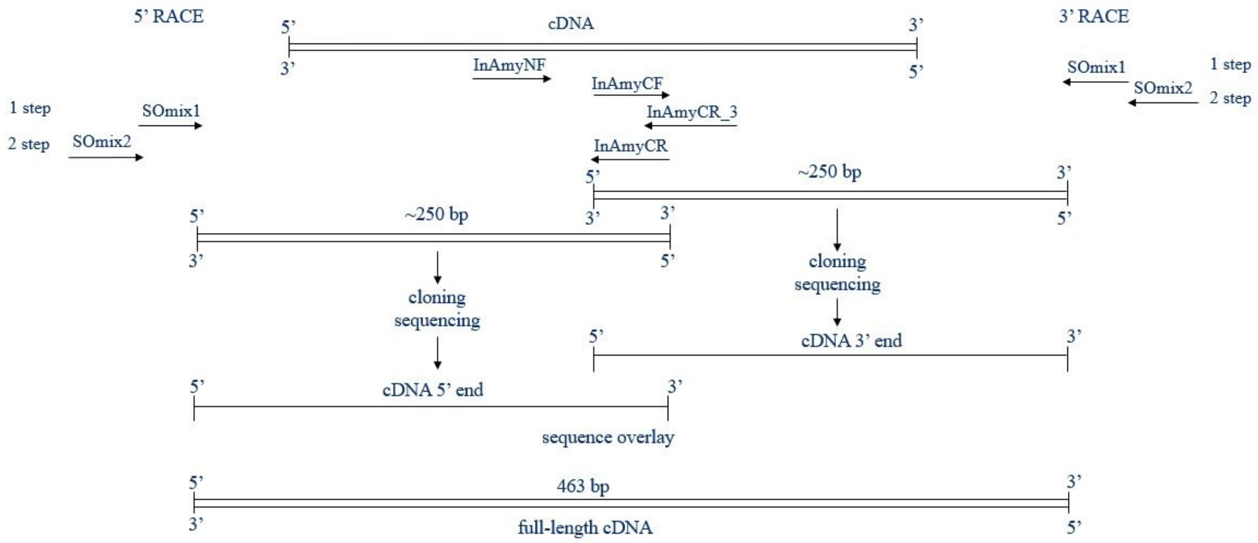

Figure S1. The RACE scheme for cDNA sequencing determination of magnificamides encoding genes.

```
DNA      tagggcaagcagtggtatcaacgcagagtacgggggaataaaccatcagtggcgaaattc

DNA      tactactcactgggagctttacttatcagcaaaATGAAAACTTTAATACTTCTGGTTGGT
protein                                    ·M··K··T··L··I··L··L··V··G·

DNA      CTTGCCCTTGTTTTCTTGGCGAGTGGAATGCCCGTGAATGAAGATCAAGAAATGAATTCT
protein  ·L··A··L··V··F··L··A··S··G··M··P··V··N··E··D··Q··E··M··N··S·

DNA      GAGGGCAACTCGTGCTATATCTACCATGGTGTGTACGGGATATGCAAGGCTAAGTGCGCG
protein  ·E··G··N··S··C··Y··I··Y··H··G··V··Y··G··I··C··K··A··K··C··A·

DNA      GAAGACGAAAAAGCCATGGCGGGAATGGGTGTGTGTGAAGGCGATCTCTGCTGTTATAAA
protein  ·E··D··E··K··A··M··A··G··M··G··V··C··E··G··D··L··C··C··Y··K·

DNA      ACACCATGGTAAtcatcggtggattttcgaccaggataatgaaatacaaaacaaataatt
protein  ·T··P··W·ter

DNA      gctaataatgcataccattaaaatcctttagag
```

Figure S2. cDNA obtained by Rapid Amplification of cDNA Ends and deduced magnificamide sequence. The exons are in uppercase letters, while the introns are in lowercase letters. The deduced amino acid residues are represented as single letter abbreviations. The signal peptide and propeptide are in bold letters and bold underlined, respectively. The sequence corresponding to magnificamide 1-1 is boxed.

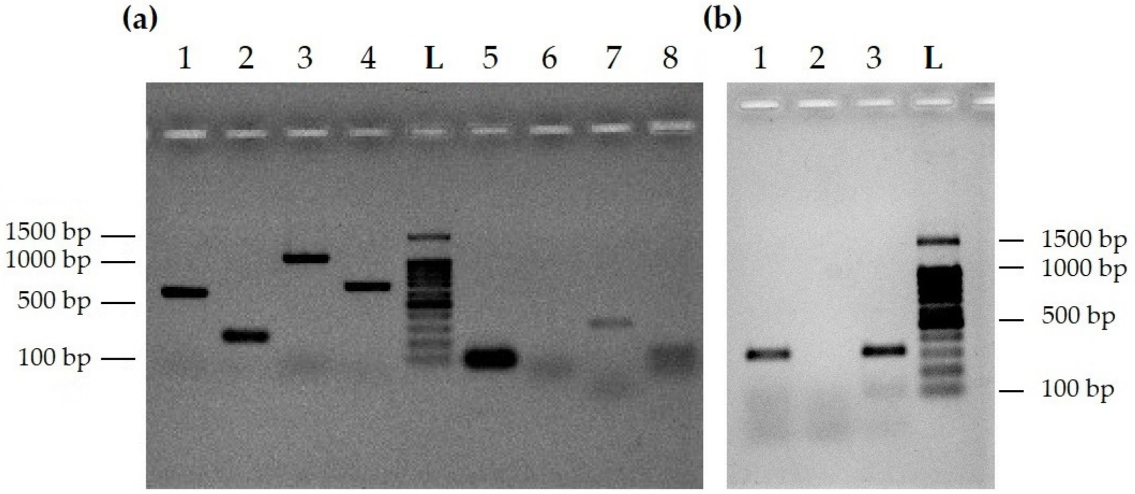

(a)	Pair of primers	Template	Flanking region	Length
1	InAmy_5UTR_out_F<br>InAmy_NF_full_R	gDNA	5′UTR, intron 1, part of signal peptide	598 bp
2	InAmy_5UTR_in_F<br>InAmy_NF_full_R	gDNA	5′UTR, intron 1, part of signal peptide	232 bp
3	HmagGAPDH_F<br>HmagGAPDH_R	gDNA	Exon-intron-exon region of *gapdh*	1084 bp
4	HmagGAPDH_intron_F<br>HmagGAPDH_intron_R	gDNA	Intron of *gapdh*	681 bp
5	InAmy_5UTR_in_F<br>InAmy_NF_full_R	cDNA	5′UTR, intron 1, part of signal peptide	232/93 bp
6	InAmy_NF_full_sig<br>InAmy_insideIn2_R	cDNA	Signal peptide, intron 2	553/0 bp
7	InAmy_insideIn3_F	cDNA	Intron 3, 3′UTR	319/0 bp
8	HmagGAPDH_F<br>HmagGAPDH_R	cDNA	Exon-intron-exon region of *gapdh*	135 bp

Figure S3. Agarose gel images of PCR products obtained by detection of intron retention with appropriate primers **(a)** (Table S1) and testing of 116 and 146 tentacle samples (lanes 1 and 3, respectively) and 146 body sample (lane 2) for the presence of a third intron retained in cDNAs **(b)**. L – 100+ bp DNA Ladder (Evrogen JSC, Russia).
